# The Brazilian version of the High-Activity Arthroplasty Score: cross-cultural adaptation

**DOI:** 10.1590/1516-3180.2023.0121.26072023

**Published:** 2023-12-08

**Authors:** Nathalia Sundin Palmeira de Oliveira, Themis Moura Cardinot, Danúbia da Cunha de Sá Caputo, Julia Ribeiro Soares, Letícia Nunes Carreras Del Castillo Mathias, Luiz Alberto Batista, Liszt Palmeira de Oliveira

**Affiliations:** I MD. Orthopedist, Master Student, Universidade do Estado do Rio de Janeiro (UERJ), Rio de Janeiro (RJ), Brazil.; II PhD. Physical Educator, Professor, Universidade Federal Rural do Rio de Janeiro (UFRRJ), Rio de Janeiro (RJ), Brazil.; III PhD. Physical Therapist, Post Doctoral Researcher, Universidade do Estado do Rio de Janeiro (UERJ), Rio de Janeiro (RJ), Brazil.; IV Undergraduate Student, Faculdade de Ciências Médicas (FCM), Universidade do Estado do Rio de Janeiro (UERJ), Rio de Janeiro (RJ), Brazil.; V PhD. Physiotherapist, Universidade do Estado do Rio de Janeiro (UERJ), Rio de Janeiro (RJ), Brazil.; VI PhD. Physical Educator, Professor, Universidade do Estado do Rio de Janeiro (UERJ), Rio de Janeiro (RJ), Brazil.; VII MD, PhD. Orthopedist, Professor, Universidade do Estado do Rio de Janeiro (UERJ), Rio de Janeiro (RJ), Brazil.

**Keywords:** Arthroplasty, Replacement, Hip, Arthroplasty, Replacement, Knee, Patient-reported outcome measure, Surveys and questionnaires, Sports, Translation, Cultural adaptation, Quality of life, Physical activity, Exercise

## Abstract

**BACKGROUND:**

The High Activity Arthroplasty Score (HAAS) is a self-administered questionnaire, developed in British English, that reliably and validly measures the levels of sports activities in patients following hip and knee arthroplasty surgery.

**OBJECTIVE:**

To cross-culturally adapt the HAAS to Brazilian Portuguese language.

**DESIGN AND SETTING:**

A cross-sectional study was conducted at a public university hospital in Brazil.

**METHODS:**

The Brazilian version of the HAAS was created through a six-step process: translation, synthesis, committee review, pretesting, back-translation, and submission to developers. The translation step was conducted by two independent bilingual translators, both native speakers of Brazilian Portuguese. The back-translation was performed by an independent translator, a native speaker of British English. To ensure the questionnaire's comprehensibility, 46 volunteers (51% men; average age 34-63) participated in the pre-testing step.

**RESULTS:**

The cross-cultural adaptation process necessitated modifications to certain terms and expressions to achieve cultural equivalence with the original HAAS.

**CONCLUSION:**

The HAAS has been translated from English into Brazilian Portuguese and culturally adapted for Brazil. The validation process for HAAS-Brazil is currently underway.

## INTRODUCTION

The functional outcome of hip and knee arthroplasty can be evaluated using health-related quality of life instruments, such as questionnaires and scales. Current literature provides instruments that primarily assess pain as the main symptom, thereby presenting a limiting factor in the performance of low-demand daily activities (DA).^1–3^

The focus on pain and DAs presents a challenge in identifying individuals who exhibit no pain limitation during low-demand activities, including DAs, but experience limitations during more strenuous activities, such as sports.^4^ Current instruments fall short in assessing significant functional differences, such as walking on uneven terrain, running, climbing stairs, and gauging the level of physical or sports performance.^4^

In response to these dilemmas, Talbot et al. developed and validated the High-Activity Arthroplasty Score (HAAS).^4^ This tool is designed to assess a patient's functional ability by incorporating a broader spectrum of physical and sporting activities, in addition to the traditional focus on painful symptoms. The HAAS is a self-administered questionnaire divided into four domains: i) *Walking;* ii) *Running;* iii) *Stair Climbing;* and iv) *Activity Level.* Each domain is designed to assess the patient's maximum capacity, resulting in a score that ranges from 0 to 18. Higher scores indicate superior patient function. The HAAS was originally developed in British English, and no cultural adaptation for Brazilian Portuguese is currently available.

## OBJECTIVE

The objective of this study was to adapt the HAAS cross-culturally from British English to Brazilian Portuguese. We hypothesized that the adaptation to Brazilian Portuguese and its subsequent application in Brazil would be both feasible and acceptable.

## METHODS

### Type of study

This is a cross-sectional, quanti-qualitative study focused on the cross-cultural adaptation of a questionnaire. The primary data was collected between September 2021 and August 2022.

The ethics committee of Hospital Universitário Pedro Ernesto, affiliated with Universidade do Estado do Rio de Janeiro (UERJ), granted approval for this study on August 30, 2021 (approval number 50529321.3.0000.5259). All participants provided their informed consent. Dr. Simon Talbot, the primary author of the HAAS, granted permission for its cross-cultural adaptation into Brazilian Portuguese on December 28, 2020.

### Cross-cultural adaptation

**Figure 1:**
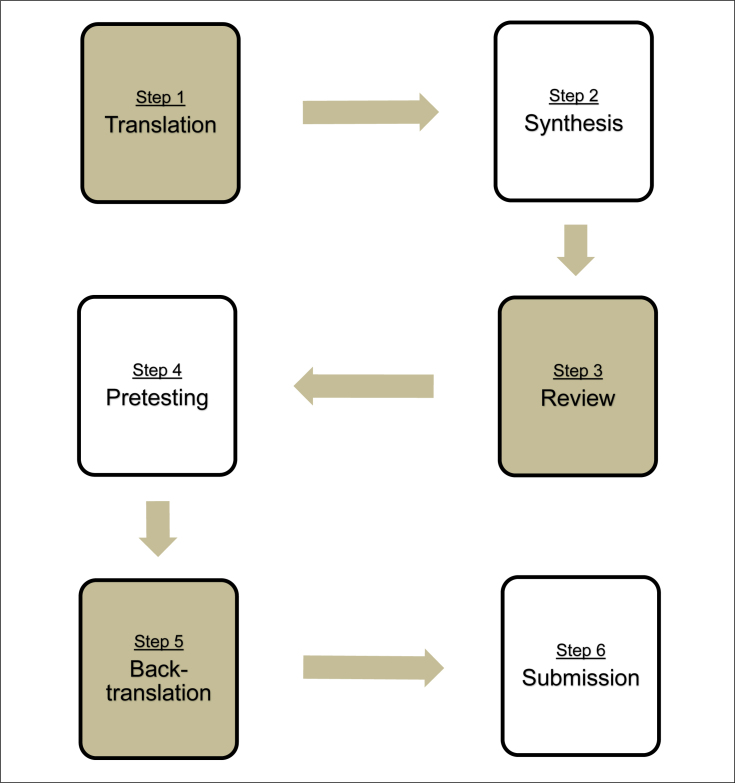
Six steps of cross-cultural adaptation: translation, synthesis, review by committee, pretesting, back-translation, and submission of documentation to the developers.

To adapt the HAAS, we adhered to the guidelines suggested by Beaton et al.^5^ with further considerations by Borsa, Damasio, and Bandeira.^6^ The procedure encompasses six steps: translation, synthesis, review by committee, pretesting, back-translation, and submission of documentation to the developers (Figure 1).

### Step 1: Translation

The HAAS was initially translated from English to Brazilian Portuguese by two independent translators, both native speakers of Brazilian Portuguese and fluent in English. This process resulted in two distinct Brazilian Portuguese blind translations: T_1_ and T_2_.

### Step 2: Synthesis

Two native Brazilian Portuguese speakers, residing in Brazil, synthesized T_1_ and T_2_ into the Brazilian Portuguese language. A reconciled version, T_1,2_, was created, and the entire process was duly documented.

### Step 3: Review by a committee

A multidisciplinary committee was formed to review T_1,2_, comprising experts in the construct under evaluation and cross-cultural adaptation studies. This committee included one physiotherapist, two orthopedists, and two physical educators. Additionally, one committee member held a degree in Language, specializing in translation and communication.

The aim of this step was to assess the semantic, idiomatic, cultural, and conceptual equivalences between the original version and T_1,2_, thereby identifying necessary adaptations. Consequently, a pretesting version (V_1_) was produced. The adaptation process was guided by the Coefficient Content Validity (CCV) proposed by Hernandez-Nieto.^7^

### Step 4: Pretesting

The objective of this step was to determine whether volunteers found the V_1_ items, instructions, and response scale comprehensible. The Three-Step Test-Interview (TSTI) employing a 5-item Likert Scale was utilized to evaluate the questionnaire's adaptation.^8,9^ The sample size was established using the saturation criteria technique.^10^

The results of the pretesting were analyzed through a qualitative assessment, taking into account suggestions for improved adaptation and comprehension from the volunteers. This process led to the creation of a final version (V_f_).

### Step 5: Back-translation

Back-translation can be utilized to assess whether the conceptual equivalence between the synthesized and revised V_f_ and the original instrument has been preserved. This process facilitates the evaluation of the culturally adapted instrument by its developers.^6^

The back-translation was conducted blindly by a native British English speaker who is fluent in Portuguese but lacks technical knowledge of the study's subject matter. The entire process was meticulously documented in writing.

### Step 6: Submission of documentation to the developers

The aim of this step was to present the Brazilian version of HAAS to the original developers.

## RESULTS

Step 1 produced two independent translations: T_1_ and T_2_ (Box 1).

**Box 1:** Step 1: Independent Translations (T1 e T2)

T1	T2
Escore de artroplastia e atividade física de alta demanda Selecione seu maior nível funcional em cada uma das quatro categorias 1 Andando (máx. 5 pontos) 5 em superfície irregular > 1 hora 4 sem limite em superfície plana, com dificuldade em superfície irregular 3 sem limite em superfície plana, incapaz de andar em superfície irregular 2 pelo menos 30 minutos em superfície plana 1 curtas distâncias sem auxílio (até 20 metros) 0 utilizando dispositivos de auxílio para andar curtas distâncias ou pior	Pontuação de Artroplastia de Alta Atividade Selecione seu nível de função mais alta em cada uma das quatro categorias 1 Caminhada (máx. 5 pontos) 5 em ladeira/terreno em subida por mais de 1 hora 4 em terreno plano sem dificuldade, mas em terreno acidentado com dificuldade 3 em terreno plano sem dificuldade, mas não consigo caminhar em terreno acidentado 2 em terreno plano por pelo menos 30 minutos 1 em distâncias curtas sem ajuda (até 20 metros) 0 usando dispositivos de apoio para distâncias curtas ou não consigo caminhar

2 Correndo (máx. 4 pontos) 4 mais que 5km 3 trotar até 5km 2 correr facilmente na rua 1 correr alguns passos para evitar trânsito, se necessário 0 não consegue correr	2 Corrida (máx. 4 pontos) 4 Corro mais de 5km 3 Corro devagar até 5km 2 Corro facilmente para atravessar a rua 1 Corro poucos passos para evitar o tráfego ao atravessar a rua, se necessário 0 Não consigo correr

3 Subindo escadas (máx. 3 pontos) 3 subir dois degraus por vez 2 subir sem apoio no corrimão 1 subir com apoio no corrimão ou bengala/muleta 0 não consegue subir escadas	3 Subir escadas (máx. 3 pontos) 3 Subo 2 degraus de cada vez 2 Subo sem apoio de corrimão 1 Subo com apoio de corrimão ou bengala 0 Não consigo subir escadas

4 Nível de atividade (máx. 6 pontos) 6 esportes competitivos. Ex.: tênis individual, correr > 10km, andar de bicicleta >80km 5 esportes sociais. Ex.: tênis em dupla, esquiar, trotar < 10km, exercícios aeróbicos de alto impacto 4 atividades recreacionais vigorosas. Ex.: montanhismo, exercícios aeróbicos de baixo impacto, jardinagem pesada, trabalho braçal / rural 3 atividades recreacionais moderadas. Ex.: golfe, jardinagem leve, atividades de trabalho leve 2 atividades recreacionais leves. Ex.: caminhadas leves, bocha 1 atividades ao ar livre apenas quando necessário. Ex.: caminhar distâncias curtas para fazer compras 0 restrito ao lar sem necessidade de auxílio	4 Nível de atividade (máx. 6 pontos) 6 Esportes competitivos, ex.: tênis simples, corrida > 10km, ciclismo >80km 5 Esportes sociais, ex.: tênis de dupla, corrida <10km, aeróbica de alto impacto 4 Atividades recreativas vigorosas, ex.: caminhada em trilhas, aeróbica de baixo impacto, jardinagem pesada ou trabalho manual/agricultura 3 Atividades recreativas moderadas, ex.: golfe, jardinagem leve, atividades leve no trabalho 2 Atividades recreativas leves, ex.: caminhadas curtas, boliche, 1 Apenas atividades ao ar livre obrigatórias, ex.: caminhar uma curta distância para fazer compras 0 Recluso em casa sem assistência

(máx. 18 pontos)	(máx. 18 pontos)

The synthesis of T_1_ and T_2_ produced T_1,2_ (Box 2), that was evaluated and reviewed by the multidisciplinary committee on the third step.

**Box 2:** Step 2: Synthesis of translations (T1,2)

Pontuação (Escore) de Artroplastia de Alta Atividade Selecione o seu maior nível funcional em cada uma das quatro categorias. 1 Caminhada (máx. 5 pontos) 5 Em terreno irregular por mais de 1 hora 4 Sem limitação em terreno plano, mas com dificuldade em terreno irregular 3 Sem limitação em terreno plano, mas não consigo andar em terreno irregular 2 Pelo menos 30 minutos em terreno plano 1 Em curtas distâncias sem ajuda (até 20 metros) 0 Usando apoio para caminhar curtas distâncias ou uma condição pior

2 Corrida (máx. 4 pontos) 4 Corro mais de 5km 3 Corro devagar (trote) até 5km 2 Correr facilmente para atravessar a rua 1 Corro poucos passos para desviar dos carros ao atravessar a rua, se necessário 0 Não consigo correr

3 Subir escadas (máx. 3 pontos) 3 Subo 2 degraus de cada vez 2 Subo sem apoiar no corrimão 1 Subo apoiando no corrimão ou na bengala/muleta 0 Não consigo subir escadas

4 Nível de atividade física (máx. 6 pontos) 6 Esportes competitivos. Exemplos: tênis simples (individual), corrida maior que 10km, ciclismo maior que 80km 5 Esportes sociais. Exemplos: tênis de dupla, esqui, corrida menor que 10km, exercícios aeróbicos de alto impacto 4 Atividades recreativas vigorosas. Exemplos: montanhismo (caminhada em trilhas), exercícios aeróbicos de baixo impacto, jardinagem pesada, trabalho braçal/rural 3 Atividades recreativas moderadas. Exemplos: golfe, jardinagem leve, atividades leves de trabalho 2 Atividades recreativas leves. Exemplos: caminhadas curtas, bocha/boliche 1 Atividades ao ar livre apenas quando necessário. Exemplos: caminhar distâncias curtas para fazer compras 0 Recluso em cada (realiza apenas tarefas do lar) sem necessidade de ajuda (máx. 18 pontos)

The main modifications proposed by the committee are listed in Table 1 and Table 2.

**Table 1:** Main modifications proposed by the committee

Original	Adapted
Competitive sports	*Esportes de alto rendimento com ênfase na competição*
Social sports	*Esportes sociais sem ênfase na competição*
Vigorous recreational activities	*Atividades físicas vigorosas*
Moderate recreational activities	*Atividades físicas moderadas*
Light recreational activities	*Atividades físicas leves*
Select	*Marque um X ou circule*
> 1 hour	*por mais de 1 hora*
e.g.	*exemplos:*

**Table 2:** Main modifications proposed about sports and physical activities

Original examples	Adapted examples	
Singles tennis/doubles tennis		
Running	*Futebol*	
Cycling	*Vôlei*	*Faxina pesada*
Jog/jogging	*Basquete*	*Trilha moderada*
Skiing	*Handebol*	*Faxina leve*
High impact aerobics	*Natação*	*Hidroginástica*
Low impact aerobics	*Tênis*	*Dança de salão*
Hill-walking	*Corrida*	*Pilates*
Heavy gardening	*Ciclismo*	*Trilha leve*
Manual work/farming	*Surfe*	*Bocha/boliche*
Golf	*Skate*	*Hidroterapia*
Light gardening	*Crossfit*	*Exercícios fisioterápicos para fortalecimento muscular*	
Light working activities	*Dança vigorosa*
Lawn bowls	*Exercício aeróbico vigoroso (bicicleta ergométrica, spinning, elíptico, esteira)*	

The qualitative analysis undertaken by the multidisciplinary committee of specialists was guided by the CCV.^7^ Items in which CCV was below 0.8 were modified by the committee prior to pretesting. Grammar, typing, and formatting errors were revised as part of this step. As a result, a version (V_1_) for pre-test was produced (Box 3).

**Box 3:** Step 3: Pre-test version (V1) of HAAS

High Activity Arthroplasty Score - Brazil Selecione o seu maior nível funcional em cada uma das quatro categorias. 1 Caminhando (máx. 5 pontos) 5 Caminho em terreno irregular por mais de 1 hora 4 Caminho sem limitação em terreno plano, mas com dificuldade em terreno irregular 3 Caminho sem limitação em terreno plano, mas não consigo caminhar em terreno irregular 2 Caminho pelo menos 30 minutos em terreno plano 1 Caminho curtas distâncias sem ajuda (até 20 metros) 0 Caminho curtas distâncias usando ou não consigo caminhar

2 Correndo (máx. 4 pontos) 4 Corro mais de 5km 3 Corro devagar até 5km 2 Corro facilmente para atravessar a rua 1 Corro poucos passos para atravessar uma rua, se necessário 0 Não consigo correr

3 Subindo escadas (máx. 3 pontos) 3 Subo 2 degraus de cada vez 2 Subo sem apoiar no corrimão 1 Subo apoiando no corrimão ou na bengala/muleta 0 Não consigo subir escadas

4 Nível de atividade física (máx. 6 pontos) 6 Pratico esportes de alto rendimento com ênfase na competição Exemplos: futebol, vôlei, basquete, natação, tênis, corrida, ciclismo, surfe, skate etc. 5 Pratico esportes socialmente sem ênfase na competição Exemplos: futebol, vôlei, basquete, natação, tênis, corrida, ciclismo, surfe, skate etc. 4 Pratico atividades físicas vigorosas Exemplos: trilha vigorosa, dança vigorosa, exercício aeróbico vigoroso (bicicleta ergométrica, spinning, elíptico, esteira), faxina pesada etc. 3 Pratico atividades físicas moderadas Exemplos: trilha moderada, faxina leve, hidroginástica, dança de salão, pilates etc. 2 Pratico atividades físicas leves Exemplos: trilha leve, bocha/boliche, hidroterapia, exercícios fisioterápicos para fortalecimento muscular 1 Pratico atividades ao ar livre apenas quando necessário. Exemplos: caminhar distâncias curtas para fazer compras 0 Estou recluso em cada (realizo apenas tarefas do lar) sem necessidade de ajuda

(máx. 18 pontos)

V_1_ was applied to 46 volunteers (51% men) with a mean age of 36-63 years old (min 19, max 69) in a heterogeneous sample regarding scholarity and income, according to data compiled in Table 3.

**Table 3:** Descriptive data of pre-test volunteers

Scholarity	Sex	Age mean (min-max)	Income	Skin Colorж	BMI (w/h^2^) mean (min-max)	Comorbidity n (%)	Physical Activity n (%)	HAAS mean (min-max)	Time for filling mean
**Middle School or less** (n = 10)		42.5 (26-69)	<3 basic salaries (n = 7)		28.6 (24.69-36.8)	3 (30%)	3 (30%)	11 (6-16)	0:04:33
	**M** (n = 5)	40.2 (26-56)	66% 1-3 basic salaries (n = 3)	**W** 1	29.65 (24.69-38.8)	0	2	12 (6-16)	0:04:28
				**B** 3					
				**P** 1					
	**F** (n = 5)	44.8 (26-69)	75% under 1 basic salary (n = 4)	**W** 0	27.56 (25.32-32)	3	1	10 (7-14)	0:04:38
				**B** 5					
				**P** 0					
**Complete High School** (n = 16)		27.0 (19-56)	<6 basic salaries (n=15)		24.27 (20.94-29.39)	5 (33%)	14 (93.33%)	14.93 (8-18)	0:02:28
	**M** (n = 8)	25.12 (19-40)	50% no income (n=8)	**W** 3	24.29 (20.94-29.39)	3	8	15.62 (14-18)	0:01:44
				**B** 1					
				**P** 4					
	**F** (n = 8)	28.87 (20-56)	62% <1 basic salary	**W** 3	24.25 (21.64-27.34)	2	6	14.25 (8-17)	0:03:13
				**B** 3					
				**P** 2					
**College Graduated** (n = 20)
		36.8 (23-65)	10% >15 basic salaries (n = 19)		26.74 (18.56-32.74)	9 (45%)	16 (80%)	14.05 (8-18)	0:02:22
	**M** (n = 10)	38.5 (23-65)	22.2% >15 basic salaries (n = 9)	**W** 7	28.04 (22.22-32.74)	4	7	13.8 (8-18)	0:02:49
				**B** 0					
				**P** 3					
	**F** (n = 10)	35.1 (25-59)	40% 3-6 basic salaries (n = 10)	**W** 5	25.43 (18.56-31.80)	5	9	14.3 (8-18)	0:01:56
				**B** 1					
**P** 4									

ж Skin color options according to *Instituto Brasileiro de Geografia e Estatística (IBGE)*'s statistics collection (Fonte: IBGE, Diretoria de Pesquisas, Coordenação de Trabalho e Rendimento, Pesquisa Nacional por Amostra de Domicílios Contínua 2012-2019);

BMI = body mass index; HAAS = high activity arthroplasty score; F = female; M = male; W = white; B = black; P = pardo; w = weight; h = height.

Among volunteers, 73.33% were engaged in physical activities (PA) (Graphic 1).

**Graphic 1:**
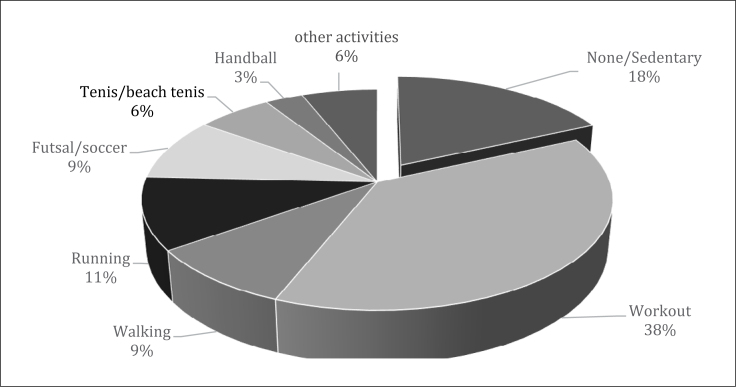
Physical activity practice and sedentary lifestyle among volunteers.

The occupations of the volunteers were as follows: students (31.11%); medical doctors (13.3%); general service assistants (13%); technical administrators (11.11%); cooks/kitchen assistants (6.66%); and other professions including professors, lawyers, security professionals, physiotherapists, laboratory technicians, marketing analysts, and retired individuals or those without an occupation (each comprising less than 5%).

Minimal modifications were proposed for the final version (V_f_), which was subsequently represented to the committee. Modifications are highlighted in Box 4.

**Box 4:** Step 4: Final version (V_f_) of HAAS with highlighted alterations

High Activity Arthroplasty Score - Brazil **Marque um X ou circule** o seu maior nível funcional em cada uma das quatro categorias. 1 Caminhando (máx. 5 pontos) 5 Caminho em terreno irregular por mais de 1 hora 4 Caminho sem limitação em terreno plano, mas com dificuldade em terreno irregular 3 Caminho sem limitação em terreno plano, mas não consigo caminhar em terreno irregular 2 Caminho pelo menos 30 minutos em terreno plano 1 Caminho curtas distâncias sem ajuda (até 20 metros) 0 Caminho curtas distâncias usando ou não consigo caminhar

2 Correndo (máx. 4 pontos) 4 Corro mais de 5km 3 Corro devagar até 5km 2 Corro facilmente para atravessar a rua 1 Corro poucos passos para atravessar uma rua, se necessário 0 Não consigo correr

3 Subindo escadas (máx. 3 pontos) 3 Subo 2 degraus de cada vez 2 Subo sem apoiar no corrimão 1 Subo apoiando no corrimão ou na bengala/muleta 0 Não consigo subir escadas

4 Nível de atividade física (máx. 6 pontos) 6 Pratico esportes de alto rendimento com ênfase na competição Exemplos: futebol, vôlei, basquete, **handebol**, natação, tênis, corrida, ciclismo, surfe, skate, **crossfit**, **lutas** etc. 5 Pratico esportes socialmente sem ênfase na competição Exemplos: futebol, vôlei, basquete, **handebol**, natação, tênis, corrida, ciclismo, surfe, skate, **crossfit**, **lutas** etc. 4 Pratico atividades físicas vigorosas Exemplos: **faxina pesada**, **jardinagem pesada/roçado/obras domésticas**, **musculação vigorosa**, **trilha vigorosa**, dança vigorosa, exercício aeróbico vigoroso (bicicleta ergométrica, spinning, elíptico, esteira), etc. 3 Pratico atividades físicas moderadas Exemplos: **faxina leve**, **jardinagem leve/pequenos reparos domésticos**, **musculação moderada**, trilha moderada, hidroginástica, dança de salão, pilates etc. 2 Pratico atividades físicas leves Exemplos: **exercícios fisioterápicos para fortalecimento muscular**, **hidroterapia**, trilha leve, bocha/boliche etc. 1 Pratico atividades ao ar livre apenas quando necessário. Exemplos: caminhar distâncias curtas para fazer compras 0 Estou recluso em cada (realizo apenas tarefas do lar) sem necessidade de ajuda (máx. 18 pontos)

Following consultation with experts, no additional pre-testing was required. The V_f_ was then back translated (Box 5) and shared with the developers for their review.^6^ They expressed satisfaction with the results and did not propose any further modifications. Thus, the V_f_ was the final translation of the HAAS, i.e., the HAAS-Brazil.

**Box 5:** Step 5: Backtranslation of HAAS-Brazil

High Activity Arthroplasty Score – Brazil Mark with an (X) your highest level of function in each of these four categories.

1 Walking (max. 5 points) 5 Walk on uneven surfaces for a period of more than 1 hour 4 Walk unrestricted on level surfaces but have trouble on uneven ground 3 Walk unrestricted on flat, level surfaces, but unable to walk on uneven ground 2 Walk for a period of at least 30 minutes on level surfaces 1 Walk short distances of up to 20 meters without requiring assistance 0 Walk short distances with assistance, or unable to walk at all

2 Running (max. 4 points) 4 Run distances more than 5 km 3 Run slowly up to distances of 5 km 2 Run easily to cross a street or intersection 1 Run a few steps to cross a street 0 No facility whatsoever to run

3 Climbing Stairs (max. 3 points) 3 Climb 2 steps at a time 2 Climb steps unassisted without handrail support 1 Climb steps but require handrail or other support, i.e., cane/crutch 0 No facility whatsoever to climb stairs

4 Level of physical activity (max. 6 points) 6 Practice high-performance sports at competition level E.g., football, volleyball, basketball, handball, swimming, tennis, running, cycling, surfing, skateboarding, crossfit, wrestling, etc. 5 Practice sports on a social basis but not at competition level E.g., football, volleyball, basketball, handball, swimming, tennis, running, cycling, surfing, skateboarding, crossfit, wrestling, etc. 4 Practice vigorous physical activity E.g., demanding house cleaning, strenuous gardening/mowing, vigorous weight training, vigorous hiking, energetic dancing, vigorous aerobic exercise, gym workouts: bike, spinning, elliptical, treadmill

3 Practice moderate physical activity E.g., light housekeeping, light gardening, moderate weight training, moderate hiking, water aerobics, ballroom dancing, pilates, etc. 2 Practice only light physical activity E.g., physical therapy exercise for muscle strengthening, hydrotherapy, light hiking, bocce/bowling, etc. 1 Participate in outdoor activities only when necessary E.g., walking short distances to the supermarket 0 I am a recluse who only performs household chores with no assistance required

(max. 18 points)

## DISCUSSION

The functional outcomes of hip and knee arthroplasty can be evaluated using health-related quality of life questionnaires and scales. However, the instruments currently available in the literature are biased by pain and DA limitation.^1–3^ Consequently, HAAS was developed and validated to assess the functional outcomes of hip and knee arthroplasty surgery in patients who do not experience significant pain or limitations in low-demand activities.^4^

Borsa et al.^6^ observed that the translation stage inherently initiates the adaptation process. This is because the subjective act of seeking words that accurately convey the intended content and construct inherently involves a degree of adaptation that a literal translation would not capture. Our understanding of translation, informed by a review of the literature, is that it is a component of the cross-cultural adaptation process. Consequently, the terminology used in the title of this paper reflects this concept.^6^

The initial phase of this study aimed at the cross-cultural adaptation of HAAS to Brazilian Portuguese, during which two translations (T_1_ and T_2_) of the original HAAS questionnaire were generated. Guillemin et al.^11^ and Beaton et al.^5^ both propose a minimum of two independent translations of the questionnaire or scale into the target language in their methodologies. The translators ideally should be bilingual, with the target language as their native language, to ensure an enhanced ability to discern the nuances and peculiarities of everyday communication within the target language.^5,6,11^ This approach enables the production of comparable translations, thereby facilitating a more effective evaluation of discrepancies and ambiguities. Furthermore, it is acknowledged that the selected translators should have varied profiles: one with a more technical understanding of the construct in question, and the other, a practitioner with a stronger emphasis on language, even if not necessarily proficient in the essence of the construct.^6^

Therefore, one of the translators who contributed to this work was an orthopedist with prior involvement in cross-cultural adaptation projects, which aimed at developing an adaptation that emphasized clinical equivalence. The second translator was a language professional with a degree in Languages and specialization in translation and communication. This ensured a translation that accurately mirrored the language used by the population, often highlighting ambiguous or excessively broad interpretations within the original questionnaire.

The second step involved merging the two translations into a single synthesized version (T_1,2_). Borsa et al.^6^ identified two potential complications at this stage: (1) a highly complex translation that may be challenging for the target population to understand, or (2) a somewhat simplistic translation that diminishes the content of the item. The research team noted that the original questionnaire's concise, simplified, and objective format could potentially confuse the target population in Brazil. This observation was considered and subsequently presented to the multidisciplinary committee of experts for further deliberation in the subsequent step.

In the third step, T_1,2_ was submitted for review to a multidisciplinary committee of specialists. This committee evaluated the structure, layout, instructions, scope, and appropriateness of the expressions within the items of the instrument, identifying any potential semantic, idiomatic, conceptual, linguistic, and contextual discrepancies between the original HAAS version and T_1,2_. This process led to several proposed structural modifications aimed at enhancing comprehension across individuals of diverse professions, educational backgrounds, income levels, and physical activity involvement.

The practice and definitions of PA are influenced by the historical context of concept formation, which can that vary based on the cultural context in which they are applied.^12,13^ Upon acknowledging that the primary objective of the original questionnaire is to assess both motor skill-related PA and sports practice as a skill, the committee suggested conceptual reframing based on available Brazilian sports literature.^12,13^ This designated a clear line of difference and hierarchy between organized/systematic sports practice and the practice of physical activities of various intensity within the domain (4) *Nível de atividade física*. Examples: “*Competitive sports*” for “*esportes de alto rendimento com ênfase na competição*” and “s*ocial sports*” for “e*sportes sociais sem ênfase na competição”* (Table 1).

The committee opted to distinguish between sports practice and PA according to energy expenditure and expected motor skill within each degree of participation. This differentiation acknowledges that there is a conceptual and practical distinction between these two modalities within the questionnaire structure. Examples: “*vigorous recreational activities*” for “*atividades físicas vigorosas*,” “*moderate recreational activities*” for “*atividades físicas moderadas*,” and “*light recreational activities*” for “*atividades físicas leves*” (Table 1). Expert consensus agreed that there was a need for modification and inclusion of examples based on the culture of the target population; removal of sports such as skiing and the inclusion of more popular sports in Brazil like surfing and soccer.

In relation to the language itself, experts proposed the full use of comparative adjectives, as well as the occurrence of abbreviations present in the original questionnaire. The questionnaire now incorporates clearer and more explanatory commands to assist the target audience in completing it accurately. Examples: “*select*” for “*marque um X ou circule*,” “*>1 hour*” for “*por mais de 1 hora*,” and “*e.g.*” for “*exemplos*” (Table 1). The proposed changes to T_1,2_ by the multidisciplinary committee of specialists, who then produced V_1_ for the pre-test step, were adhered to by the quantitative criterion of the CCV.^7^

Borsa et al.^6^ recommended conducting the pre-test with the target population, whereas the typical approach, as suggested by Guillemin et al.^11^ and Beaton et al.^5^, involves using healthy volunteers for this stage. Cross-cultural adaptation proponents have historically advocated for conducting pre-tests beyond the scope of the target population. ^1–3,14^ Given these perspectives, the decision was made to conduct the pre-test with volunteers.

Traditional empirical methodology suggests a minimum sample size of 30 to 40 volunteers for the pre-test. In this study, volunteers were consecutively selected using the saturation sampling technique. Saturation sampling, a qualitative research method, involves halting the inclusion of new participants when the data starts to show redundancy and is deemed irrelevant for further data collection by the research team.

In this study, we applied saturation sampling, which resulted in a heterogeneous group that aptly represented the Brazilian population's diversity in terms of age, education, and socio-cultural aspects. This approach adhered to the classic methodology proposed by Guillemin et al.^11^ and Beaton et al.^5^ Following the saturation sampling technique,^10^ the recruitment of new volunteers ceased when no substantial or additional contributions were discernible within the data. This cessation point was reached with a total of 46 volunteers. We incorporated the TSTI with a 5-item Likert scale into the pre-test to assess the cultural adaptation of the questionnaire.^9^

Following the initial pre-test, the researchers incorporated several modifications suggested by the volunteers and resubmitted the revised version to the expert committee. A subsequent pre-test was deemed unnecessary as no significant conceptual or structural changes were proposed.^6^ Within the TSTI methodology, the active pursuit of critique frequently elicited suggestions that had not been questioned during the examiner's passive assessment of topics. However, on certain occasions, these suggestions, when offered as solutions, risked misrepresenting the intent of a self-administered, objective, and generic questionnaire designed to evaluate the construct of interest.

The fifth step involved a back-translation, a role that has been somewhat debated within the cross-cultural adaptation process.^6^ The objective was not to achieve a literal equivalence between an adapted version and original versions but rather to maintain conceptual equivalence.^6^ Despite the debate, we acknowledge that back-translation is an effective tool for communicating and presenting the adapted instrument to the original developers. Consequently, we conducted back-translation as the fifth step, as recommended by Borsa et al.^6^ This approach contrasts with the classical methodology of Beaton et al.^5^, which positions this step after the synthesis.

The back-translation step was successfully completed, and the results were presented to the developers. They expressed satisfaction with the outcomes and did not suggest any additional recommendations. This marked the conclusion of the sixth and final step in the cross-cultural adaptation process of HAAS into Portuguese, culminating in the creation of HAAS-Brazil.

A notable limitation of this study is the execution of the pre-test, which relied on a sample from a single urban center within Brazil. It is important to acknowledge that Brazil, being a continental country, encompasses numerous regional linguistic and cultural differences. To mitigate this limitation, we attempted to assemble a diverse sample of volunteers, considering variables such as education and financial income.

## CONCLUSION

The HAAS was translated into Brazilian Portuguese and adapted to the cultural context of Brazil. Our hypothesis that this adaptation is feasible and acceptable in Brazil has been largely corroborated. However, we acknowledge that the validation of the HAAS in Brazil is still ongoing.
